# Evaluating the Impact of Trauma on Carpal Tunnel Syndrome Onset and Postsurgical Recovery: A Retrospective Study in Orthopedic Practice

**DOI:** 10.1016/j.jhsg.2025.100774

**Published:** 2025-06-19

**Authors:** Ashley A. Ellingwood, Uzoma Nwakibu, Maya Sternberg, Obinwanne Ugwonali

**Affiliations:** ∗ Peachtree Orthopedics, Atlanta, GA; † Wellstar Kennestone Regional Medical Center, Marietta, GA

**Keywords:** Carpal tunnel syndrome, Chart review, Postsurgical outcomes, Traumatic injury

## Abstract

**Purpose:**

The purpose of this study was to investigate the relationship between previous trauma to upper extremities and the subsequent development of carpal tunnel syndrome (CTS). By comparing cases of traumatically induced CTS with idiopathic CTS, the study aims to evaluate differences in symptom severity and postsurgical treatment outcomes. Additionally, the study explores other factors associated with higher pain scores and longer lengths of care.

**Methods:**

A retrospective study was conducted on patients who underwent carpal tunnel release surgery by a single hand surgeon from January 1, 2014 to October 1, 2020. Pre- and postoperative care duration and pain levels, assessed using a visual analog scale, were the primary outcomes. To analyze the predictors of pain levels and recovery time, unadjusted means were generated, followed by the development of a model to account for potential correlations and contributions.

**Results:**

The study included 478 patients (324 women and 154 men) with a mean age of 53.2 years, all of whom underwent carpal tunnel release surgery. Patients with traumatic injuries had higher initial and final pain scores compared with those with nontraumatic causes. Notably, trauma was not a significant factor in the length of care model, despite its association with pain scores. Meanwhile, worker’s compensation was a factor associated with prolonged length of care.

**Conclusions:**

This study demonstrates that traumatic injuries and other factors, such as worker’s compensation, gender, and race/ethnicity, significantly influence both pain levels and length of care in patients undergoing carpal tunnel release surgery. These findings emphasize that CTS can develop after trauma and is not exclusively idiopathic. Addressing various clinical and socioeconomic factors is essential for enhancing treatment effectiveness and managing expectations following surgery.

**Type of study/level of evidence:**

Therapy/Prevention, Etiology/Harm; IV.

Carpal tunnel syndrome (CTS) is a compressive neuropathy affecting the median nerve at the level of the wrist, characterized by an increased pressure within the carpal tunnel that leads to median nerve compression and gradual dysfunction. This condition commonly presents with pain, numbness, and tingling in the radial three and half digits and can result in motor dysfunction in severe cases. Nonsurgical management includes the use of nonsteroidal anti-inflammatory drugs, wrist splints, and steroid injections. For cases refractory to conservative measures, surgical intervention is considered and involves the release of the transverse carpal ligament to reduce pressure within the carpal tunnel.^1^

Although CTS is frequently idiopathic, various risk factors have been associated with its development. These include mechanical trauma, repetitive use of the flexor muscles, vibration exposure, and medical conditions like tenosynovitis or pregnancy, which increase the volume of other contents of the carpal tunnel, exerting more pressure on the median nerve. Other diseases and behaviors, such as diabetes, alcoholism, or vitamin deficiencies, can lead to physiological changes within the median nerve itself, ultimately lowering the threshold for permanent nerve damage after insult. Despite these known causes, research on the differences between traumatic and nontraumatic CTS remains limited.^1^, ^2^, ^3^ Researchers have shown that traumatic events, such as automobile collisions and distal radius fractures, may be associated with CTS.^4^^,^^5^ In 2006, Ettema et al^6^ identified that shearing injuries could lead to fibrosis in subsynovial connective tissue, potentially compressing the median nerve. Despite the unspecified etiology of these shearing injuries, their impact on CTS development underscores the need to delve further into the relationship between trauma and CTS.

We acknowledge that trauma or prior surgery on the upper extremities may influence an individual’s experience of pain and could impact their perception of improvement. Sun et al^7^ conducted a study in 2019 revealing that factors such as psychological distress, pain catastrophizing, and illness perceptions independently contribute to the self-reported severity of CTS. Given that our assessment includes self-reported preoperative and postoperative pain levels, it is important to consider how psychological distress stemming from trauma or desensitization because of prior surgery may either heighten or diminish the pain threshold.

In this study, we investigate the relationship between trauma to the upper extremities and the subsequent development of CTS. Our primary objective was to compare the recovery process of traumatically induced CTS to that of idiopathic CTS. We assess factors such as preoperative and postoperative length of care and changes in reported pain levels before and after surgery. In addition to this primary comparison, we also explore other demographic and clinical factors associated with higher pain scores and prolonged length of care. To conduct this analysis, we studied patients who were diagnosed with CTS and underwent surgical intervention and compared patients with and without a recent reported history of trauma to the upper extremities.

## Materials and Methods

### Patient selection

A retrospective chart review was performed on collected information of patients treated surgically for CTS by a single fellowship-trained hand, shoulder, elbow, and microvascular hand surgeon within a private orthopedic group from January 1, 2014 to October 1, 2020. Approval was given for our protocol, and the institutional review board provided oversight. The local institutional review board determined this study to be exempt. All data were deidentified; therefore, individual subject consent was not necessary. Inclusion criteria for patients included in the current study are as follows: a clinical diagnosis of CTS confirmed through clinical evaluation and EMG and documented surgical release reported in electronic medical record Athena. Patients were excluded from the study if they had a previous surgical release by another physician or previous release by the same surgeon but outside the specified time frame.

### Data collection

We present descriptive statistics of demographic and clinical variables to provide an overview of a cohort of individuals with CTS used in this study (Table 1, Table 2). Demographic and clinical variables included in each model were age, gender, race/ethnicity, workers compensation status, body mass index, diabetes history, smoking status, rheumatoid arthritis, cervical spine diagnosis, upper-extremity diagnosis, trauma, previous cervical spine or upper-extremity surgery, as well as whether the measurement of the visual analog scale (VAS) pain score or length of care was made preoperative or postoperative (Table 2, Table 3). Demographic factors, such as gender, race, and ethnicity, are recorded based on the patient’s self-identification as documented in the medical record. Additionally, multiple surgical techniques were used, including open, endoscopic, and endoscopic converted to open. Surgical approach was included as a variable in the model to account for its potential effect on pain and care duration. Clinical variables included prior upper-extremity surgery and a history of trauma, which were recorded separately; prior surgery was defined as any surgical intervention, whereas trauma referred to a documented injury. For patients who underwent bilateral carpal tunnel release, procedures were generally performed sequentially, and some had them performed simultaneously. The time interval between procedures varied across patients and was not standardized.

**Table 1:** Demographic Characteristics of Patients Diagnosed With CTS

Variable	n	Percentage
Female	324	67.8
Workers compensation	151	31.6
History of diabetes	58	12.1
History of rheumatoid arthritis	14	2.9
Body mass index (≥30 kg/m^2^)	224	46.9
Smoking status		
Never smoked	338	71.15
Current smoker	39	8.2
Former smoker	98	20.6
Race/ethnicity		
Non-Hispanic White	207	43.3
Non-Hispanic Black	135	28.2
Mexican American or Hispanic	46	9.6
Unknown	76	15.9
Other	14	3

**Table 2:** Clinical Profile of Patients Diagnosed With CTS

Variable	n	Percentage
Previous cervical spine surgery	17	3.6
Both sides have a CTS diagnosis	182	38.1
Cervical spine diagnosis	87	18.2
Upper extremity diagnosis		
None	87	18.2
Only one side	281	58.8
Both sides	110	23
Trauma		
None	239	50
Only one side	218	45.6
Both sides	21	4.4
Mechanism of injury		
Fall	62	13
Crush	31	6.5
Lift	13	2.7
Push/Pull	10	2.1
MVA	96	20.1
Hyperextension	3	0.6
Laceration	10	2.1
Unknown	11	2.3
Twisting	2	0.4
Immobilization	1	0.2
Previous upper extremity surgery		
None	380	79.5
Only one side	94	19.7
Both sides	4	0.8
Side for surgery		
Only left	130	27.2
Only right	226	47.3
Both	122	25.5

**Table 3:** Previous Upper Extremity Surgery and Nature of Injury Overview for All Patients

Status	Overall		Patients who Underwent a Bilateral CTR	
	Patients	Percentage	Patients	Percentage
No UE Hx and nontraumatic	194	40.6	90	73.8
No UE Hx and traumatic	186	38.9	21	17.2
UE Hx and nontraumatic	45	9.4	9	7.4
UE Hx and traumatic	53	11.1	2	1.6

UE Hx, upper extremity history.

Traumatic origin of CTS was determined by retrospective chart review and defined as the onset of symptoms following an identifiable event, which led the patient to seek medical care. These events included falls, crush injuries, lifting, pushing/pulling, motor vehicle accidents, hyperextension, lacerations, twisting, or immobilization, as detailed in Table 2. If no specific injury was documented, the trauma was classified as “unknown.” For motor vehicle accidents, patients were included regardless of whether fractures were presents, as long as the trauma was associated with the development of CTS symptoms. Trauma was considered if related to the surgical side, typically at the level of the wrist or hand, although this was not specifically noted during the chart review. Regarding timing, patients were classified as having a traumatic injury if presented with symptoms at the time of the injury or developed symptoms during treatment for the injury. Although there was no specific time frame set, trauma had to be documented as preceding the onset of CTS symptoms.

We primarily assessed preoperative and postoperative length of care along with reported pain levels following the preferred reporting items for systematic reviews and meta-analyses guidelines. Preoperative length of care was determined by measuring the time between the initial clinic visit for diagnosis and the day of surgery. Postoperative length of care was determined by measuring the time between the date of surgery and the final clinic visit. Pain levels were assessed using a VAS score, running from 0 (no pain) to 10 (worst pain). This was administered by a clinical assistant during all pre- and post-surgery clinic visits. Pain levels at diagnosis and at the final clinic visit were documented for each patient. For patients with bilateral CTS, a VAS score was collected per extremity. For patients with a history of upper extremity trauma, VAS scores were taken from the first visit during which CTS symptoms were reported. As this was a retrospective study, pain attribution was based on clinical notes, and we could not further distinguish pain because of CTS from other potential sources. Furthermore, the treating hand surgeon classified each patient’s pain severity as mild (VAS 0–3), moderate (4–6), or severe (7–10). This stratification is consistent with prior studies that have validated and applied similar cutoffs.^8^^,^^9^

To identify key factors influencing pain levels and length of stay, we compared unadjusted means of these outcomes across various predictor variables at each time point (pre- and postoperative) using generalized estimating equations (GEE) with a normal link and an exchangeable working correlation matrix. This method allowed us to estimate means and perform statistical inference while addressing within–subject correlations stemming from individuals contributing data from both left and right sides. Additionally, because of distributional assumptions, a log transformation was applied to the length of stay. Results for the length of care have been back-transformed to provide the geometric means.

To inform clinical decisions and patient care, we further developed a model using GEE with a normal link and an exchangeable working correlation matrix to examine variables associated with pain levels and log-transformed length of stay. Given that individuals may experience symptoms in both hands and have two measurements (pre- and postoperative), the GEE approach enables us to account for within–subject correlations over time while also accommodating potential contributions from multiple hands. We used robust standard errors that can protect against potential misspecifications in the working correlation structure. To develop the final model, we used backward elimination, sequentially removing variables based on a significance threshold of .05. By integrating longitudinal and repeated measures data, along with robust standard errors, we can identify influential factors contributing to pain levels and length of stay in individuals with CTS while controlling for multiple risk factors.

## Results

### Bivariate results

**Figure 1:**
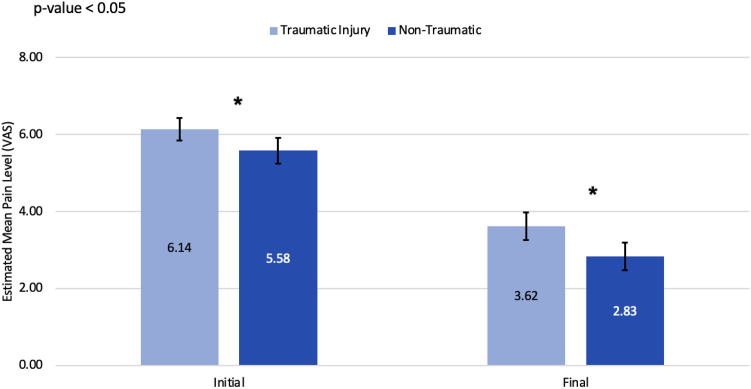
Comparing estimated mean initial and final pain levels (VAS score) between traumatic and nontraumatic injuries. Light blue bars represent traumatic injury, whereas dark blue represents nontraumatic. “∗” indicates a statistically significant difference between the two means, with a *P* < .05.

**Figure 2:**
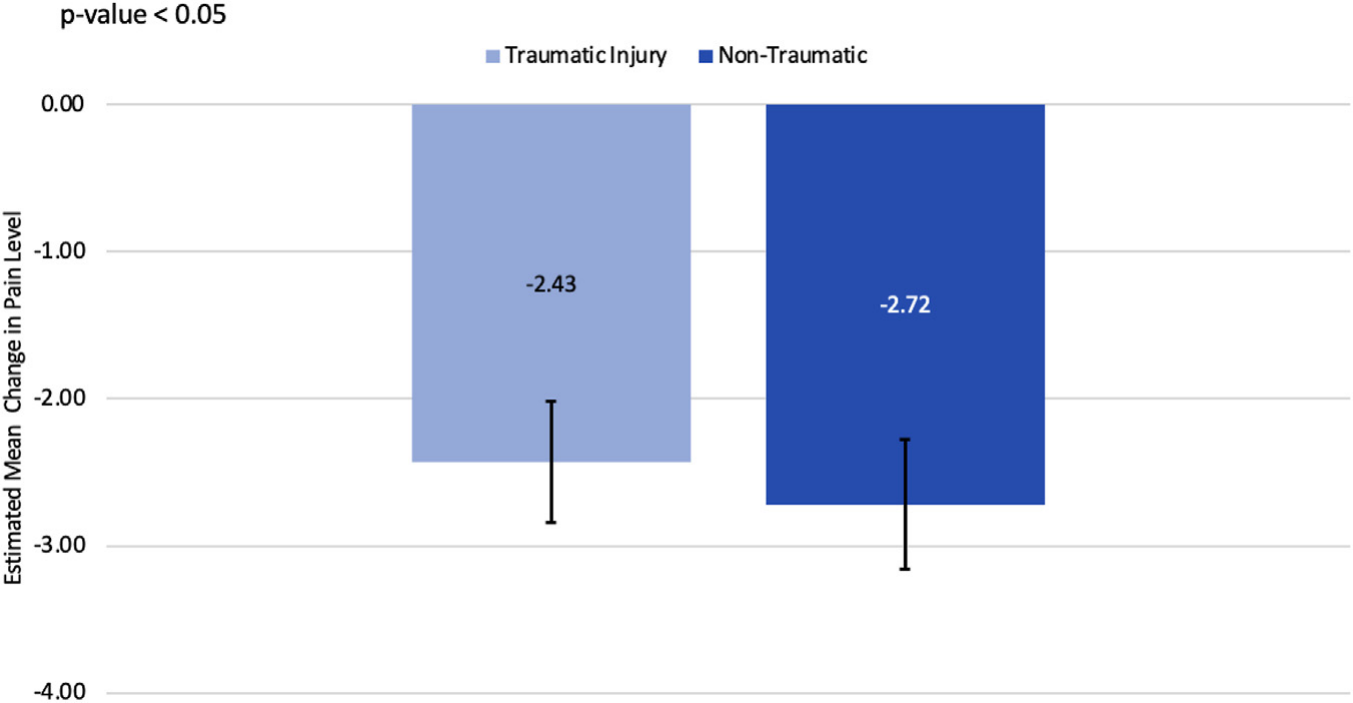
Comparing estimated mean change in pain level from the initial to the final consultation between traumatic and nontraumatic injuries. Light blue bars represent traumatic injury, whereas dark blue represents nontraumatic ones.

A total of 478 patients (324 women and 154 men) with a mean age of 53.23 ± 11.45 years and a body mass index of 30.77 ± 6.73 underwent carpal tunnel release surgery from January 1, 2014 to October 1, 2020 (Table 1). Significant associations were found between various demographic and clinical variables and both pre- and postoperative pain levels (Table 4). Unadjusted means estimated from a GEE model, incorporating trauma as the sole predictor, showed that patients with traumatic injuries reported higher initial (6.14 vs 5.58, *P* = .023) and final (3.62 vs 2.83, *P* = .003) VAS pain scores compared to those with nontraumatic CTS (Fig. 1). However, after adjusting for time, there was no statistically significant difference in the reduction of pain levels between the two groups (−2.43 vs −2.72, *P* = .397; Fig. 2). Additionally, gender, workers’ compensation status, race/ethnicity, and previous upper extremity surgery significantly influenced pain scores. Women and those on workers' compensation reported higher pain levels both before and after surgery, whereas those who had previous upper extremity surgery reported lower preoperative pain levels.

**Table 4:** Average VAS Pain Score Levels and 95% CI at Baseline and at Last Consult

Variable	Level	Mean Pain Level Preoperative (95% CI)	*P* Value (Before)	Mean Pain Level Postoperative (95% CI)	*P* Value (After)
All subjects	Overall	5.9 (5.6–6.1)	NA	3. (2.9–3.4)	NA
Trauma	No	5.6 (5.3–5.9)	.023	2.8 (2.5–3.2)	.003
	Yes	6.1 (5.9–6.4)	.	3.6 (3.3–4.0)	.
Previous upper-extremity surgery	No	6.0 (5.8–6.3)	.014	3.2 (3.0–3.5)	.712
	Yes	5.3 (4.7–5.8)	.	3.1 (2.6–3.6)	.
Cervical Spine Diagnosis	No	5.8 (5.5–6.0)	.050	3.2 (2.9–3.5)	.967
	Yes	6.3 (5.8–6.9)	.	3.2 (2.6–3.8)	.
Obesity	No	5.7 (5.4–6.0)	.209	3.2 (2.9–3.5)	.912
	Yes	6.0 (5.7–6.4)	.	3.2 (2.9–3.6)	.
Diabetes	No	5.8 (5.6–6.1)	.690	3.2 (3.0–3.5)	.887
	Yes	6.0 (5.4 – 6.6)	.	3.2 (2.4–3.9)	.
Rheumatoid arthritis	No	5.8 (5.6 – 6.1)	.070	3.2 (2.9–3.5)	.403
	Yes	6.8 (5.9–7.7)	.	3.9 (2.3–5.6)	.
Upper-extremity diagnosis	Both sides	5.8 (5.3–6.3)	.471	2.8 (2.3–3.3)	.092
	None	6.0 (5.5 – 6.6)	.	2.9 (2.3–3.5)	.
	Opposite side	5.5 (5.0 – 6.0)	.	3.4 (2.5–4.3)	.
	Same side	5.8 (5.6–6.1)	.	3.5 (3.2–3.8)	.
Gender	Female	6.1 (5.8–6.4)	.005	3.4 (3.1–3.7)	.011
	Male	5.4 (4.9–5.8)	.	2.8 (2.3–3.2)	.
Workers’ compensation	No	5.7 (5.4–6.0)	.011	2.8 (2.5–3.0)	< .0001
	Yes	6.3 (5.9–6.6)	.	4.2 (3.8–4.6)	.
Race/ethnicity	Mexican American or Hispanic	6.0 (5.3–6.8)	.0001	3.6 (2.9–4.4)	< .0001
	Non-Hispanic Black	6.5 (6.1–6.9)	.	4.0 (3.5–4.5)	.
	Non-Hispanic White	5.2 (4.9–5.8)	.	2.5 (2.2–2.8)	.
	Other	5.9 (4.6–7.4)	.	3.9 (2.3–5.6)	.
	Unknown	6.3 (5.7–6.8)	.	3.5 (2.8–4.1)	.
Smoking status	Former	5.8 (5.3–6.3)	.351	3.3 (2.8–3.9)	.863
	Heavy	5.9 (5.0–6.9)	.	3.3 (2.1–4.4)	.
	Light	6.1 (4.1–8.1)	.	4.4 (2.5–6.3)	.
	Moderate	7.3 (6.3 – 8.4)	.	3.2 (1.6–4.7)	.
	Never	5.9 (5.6–6.1)	.	3.2 (2.9–3.5)	.
Current smoking status	No	5.8 (5.6–6.1)	.228	3.2 (2.9–3.5)	.755
	Yes	6.3 (5.6–7.0)	.	3.3 (2.5–4.2)	.
Mechanism of injury	Crush	6.7 (5.9–7.4)	.063	4.4 (3.0–5.9)	.021
	Fall	6.5 (6.1–6.8)	.	3.6 (3.0–4.2)	.
	MVA	6.0 (5.5–6.6)	.	3.0 (2.5–3.6)	.
	No trauma	5.5 (5.2–5.8)	.	2.9 (2.5–3.2)	.
	Other	5.9 (5.1–6.8)	.	3.9 (3.0–4.8)	.
	Unknown	7.0 (6.0–8.0)	.	4.8 (3.3–6.4)	.
Side for surgery	Both sides	5.8 (5.4–6.3)	.975	2.7 (2.2–3.2)	.043
	Only left	5.9 (5.5–6.4)	.	3.4 (3.0–3.9)	.
	Only right	5.9 (5.5–6.2)	.	3.4 (3.0–3.8)	.
CTS diagnosis	Both sides involved	5.9 (5.5–6.3)	.982	2.8 (2.4–3.2)	.007
	One side	5.9 (5.6–6.2)	.	3.5 (3.2–3.8)	.

*P* values are based on a score test derived from a GEE model with a normal link function and an exchangeable working correlation matrix using robust standard errors.

**Figure 3:**
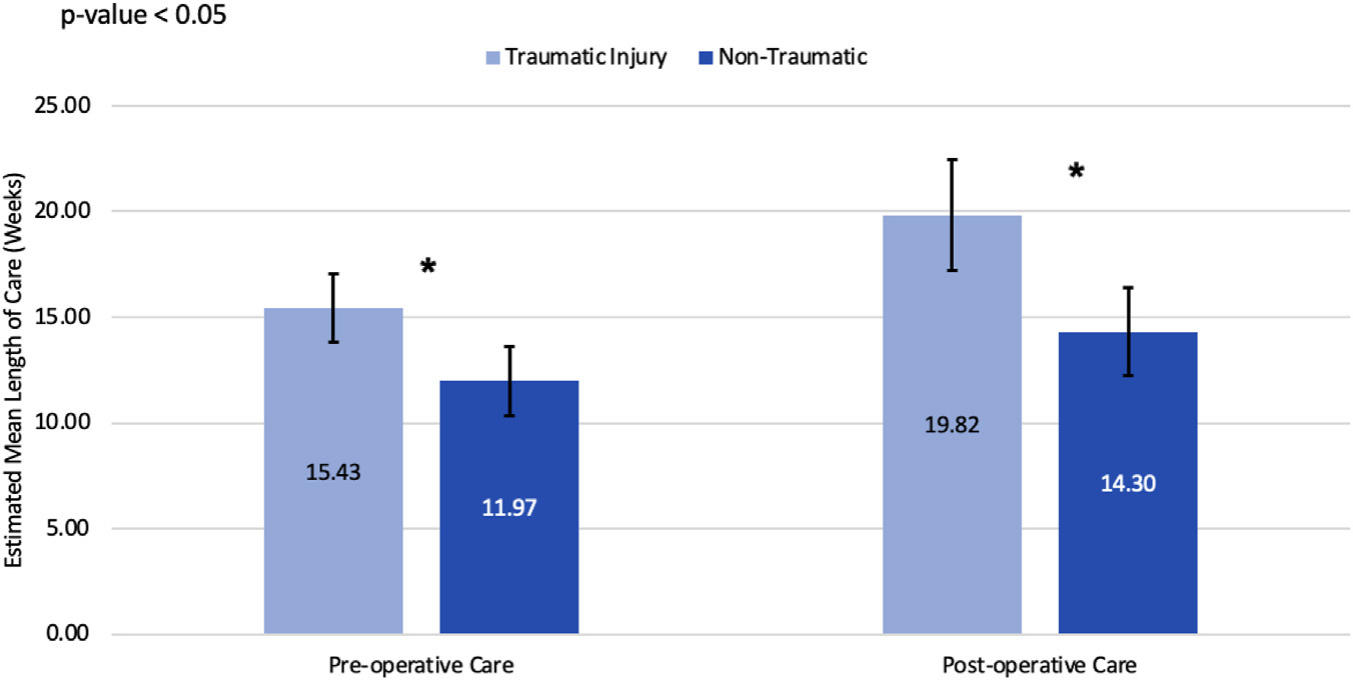
Comparing estimated mean preoperative and postoperative length of care (in weeks) between traumatic and nontraumatic injuries. Light blue bars represent traumatic injury, whereas dark blue represents nontraumatic. “∗” indicates a statistically significant difference between the two means, with a *P* < .05.

Trauma history, cervical spine diagnosis, race/ethnicity, and workers’ compensation were associated with length of care (Table 5). Patients with traumatic injuries had a longer preoperative (15.43 vs 11.97 weeks, *P* = .007) and postoperative length of care (19.82 vs 14.3 weeks, *P* = .003; Fig. 3). Although patients with a cervical spine diagnosis experienced longer preoperative care periods, race/ethnicity and workers’ compensation were associated with both longer pre- and postoperative lengths of care.

**Table 5:** Geometric Mean Length of Care and 95% CI Before and After Surgery

Variable	Levels	Geometric Mean Pre-op Length of Care Preoperative	*P* Value (Pre-op)	Geometric Mean Post-op Length of Care Postoperative	*P* Value (Post-op)
All subjects	Overall	13.6 (12.3–14.9)	NA	16.7 (15.0–18.6)	NA
Trauma	No	12.0 (10.3–13.9)	.007	14.3 (12.2–16.7)	.003
	Yes	15.4 (13.8–17.2)	.	19.8 (17.2–22.9)	.
Previous upper-extremity surgery	No	13.5 (12.2–15.0)	.885	16.6 (14.8–18.6)	.718
	Yes	13.7 (11.0–17.2)	.	17.4 (13.7–22.2)	.
Cervical spine diagnosis	No	12.7 (11.4–14.1)	.001	16.2 (14.4–18.2)	.147
	Yes	18.3 (15.2–22.0)	.	19.6 (15.6–24.6)	.
Obesity	No	13.2 (11.7–15.0)	.625	17.0 (14.8–19.6)	.762
	Yes	13.9 (12.0–16.0)	.	16.5 (14.1–19.2)	.
Diabetes	No	13.6 (12.3–15.0)	.755	17.2 (15.4–19.2)	.204
	Yes	13.0 (9.7–17.4)	.	13.9 (10.2–18.9)	.
Rheumatoid arthritis	No	13.5 (12.3–14.9)	.997	17.0 (15.3–18.9)	.132
	Yes	13.6 (6.3–29.1)	.	10.6 (6.0–18.5)	.
Upper-extremity Diagnosis	Both sides	16.6 (13.5–20.4)	.0003	16.9 (13.6–20.9)	.083
	None	8.6 (6.9–10.7)	.	12.8 (9.9–16.5)	.
	Opposite side	15.7 (11.1–22.0)	.	14.7 (8.9–24.4)	.
	Same side	14.3 (12.8–16.0)	.	18.6 (16.3–21.3)	.
Gender	Female	13.8 (12.3–15.4)	.627	17.9 (15.8–20.4)	.059
	Male	13.1 (10.9–15.6)	.	14.5 (12.2–17.3)	.
Workers comp	No	12.1 (10.7–13.7)	.0002	12.8 (11.3–14.5)	< .0001
	Yes	17.3 (15.1–19.7)	.	30.3 (26.4–34.7)	.
Race/ethnicity	Mexican American or Hispanic	12.8 (9.8–16.8)	.035	20.7 (15.3–27.9)	< .0001
	Non-Hispanic Black	16.5 (13.9–19.5)	.	23.9 (19.7–28.9)	.
	Non-Hispanic White	11.6 (9.9–13.6)	.	12.0 (10.3–13.9)	.
	Other	17.7 (11.3–27.7)	.	24.2 (12.1–48.5)	.
	Unknown	14.3 (11.4–17.5)	.	18.7 (14.8–23.7)	.
Smoking status	Former	12.3 (9.9–15.4)	.661	16.0 (12.4–20.7)	.605
	Heavy	16.2 (11.1–23.6)	.	13.3 (8.8–20.3)	.
	Light	8.0 (2.1–30.2)	.	25.6 (10.0–65.5)	.
	Moderate	12.2 (7.0–21.1)	.	21.8 (12.7–37.3)	.
	Never	14.0 (12.5–15.6)	.	17.0 (15.1–19.1)	.
Current smoking status	No	13.6 (12.3–15.0)	.904	16.7 (15.0–18.7)	.946
	Yes	13.9 (10.1–19.0)	.	16.6 (12.0–22.9)	.
Mechanism of injury	Crush	16.4 (12.4–21.7)	.02	22.0 (13.6–35.7)	.011
	Fall	15.0 (12.1–18.6)	.	24.7 (19.1–32.1)	.
	MVA	15.3 (12.8–18.4)	.	16.0 (12.9–20.0)	.
	No trauma	12.0 (10.3–13.9)	.	14.3 (12.3–16.8)	.
	Other	17.7 (13.8–22.8)	.	21.0 (15.2–28.9)	.
	Unknown	10.2 (7.7–13.4)	.	20.4 (10.1–41.4)	.
Side for surgery	Both sides	13.7 (11.4–16.5)	.693	16.5 (13.4–20.3)	.964
	Only left	14.3 (11.9–17.3)	.	16.6 (13.7–20.1)	.
	Only right	13.0 (11.4–14.8)	.	17.0 (14.7–19.8)	.
CTS diagnosis	Both sides involved	14.3 (12.2–16.7)	.363	15.3 (12.9–18.2)	.177
	One side	13.0 (11.6–14.7)	.	17.8 (15.7–20.2)	.

*P* values are derived from a score test within a GEE model using a normal link function and an exchangeable working correlation matrix following a log transformation. Reported estimates represent back-transformed geometric means. Robust standard errors are used.

### Model results

**Figure 4:**
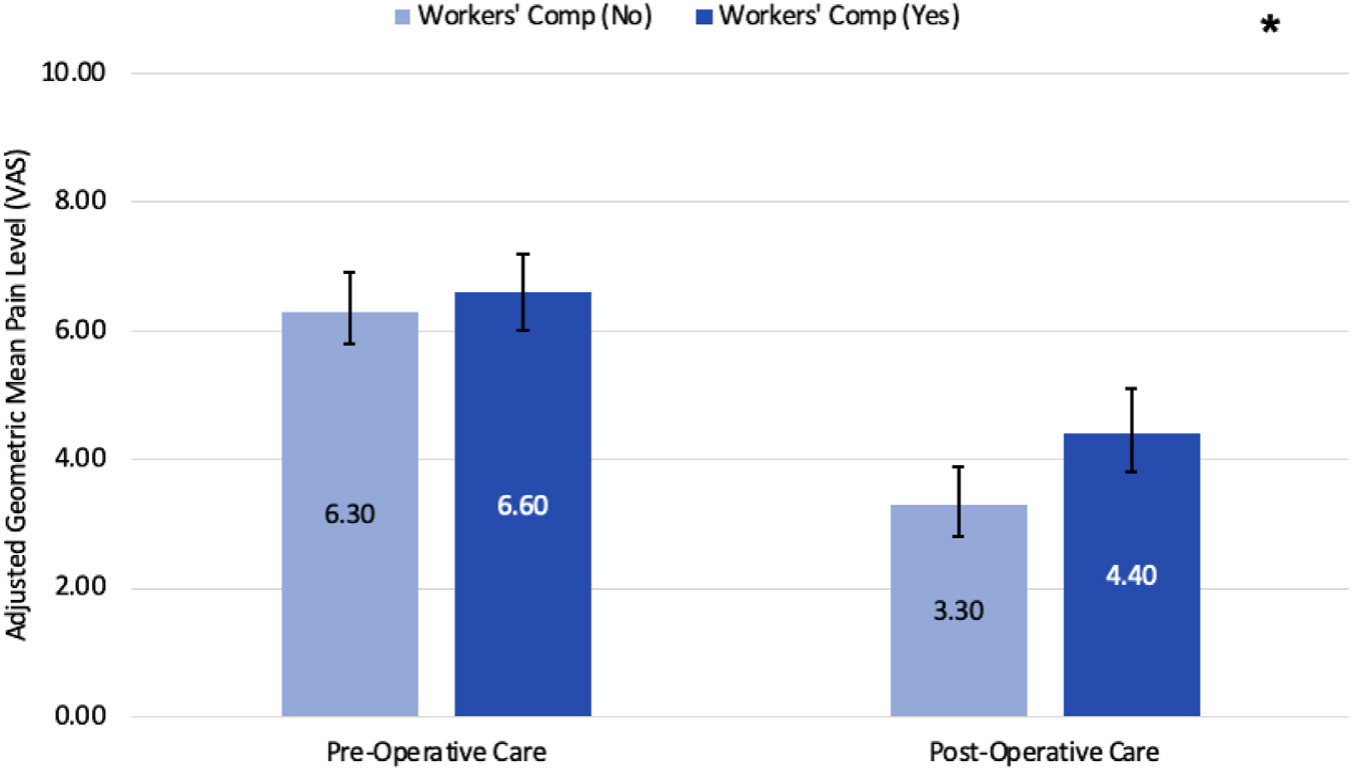
Comparing estimated adjusted geometric means and 95% confidence interval (CI) for the interaction between period (preop vs postop) and workers’ compensation regarding pain level (VAS score). Light blue bars represent no workers’ compensation, whereas dark blue represents workers’ compensation. “∗” indicates a statistically significant difference between the two means, with a *P* < .05.

**Figure 5:**
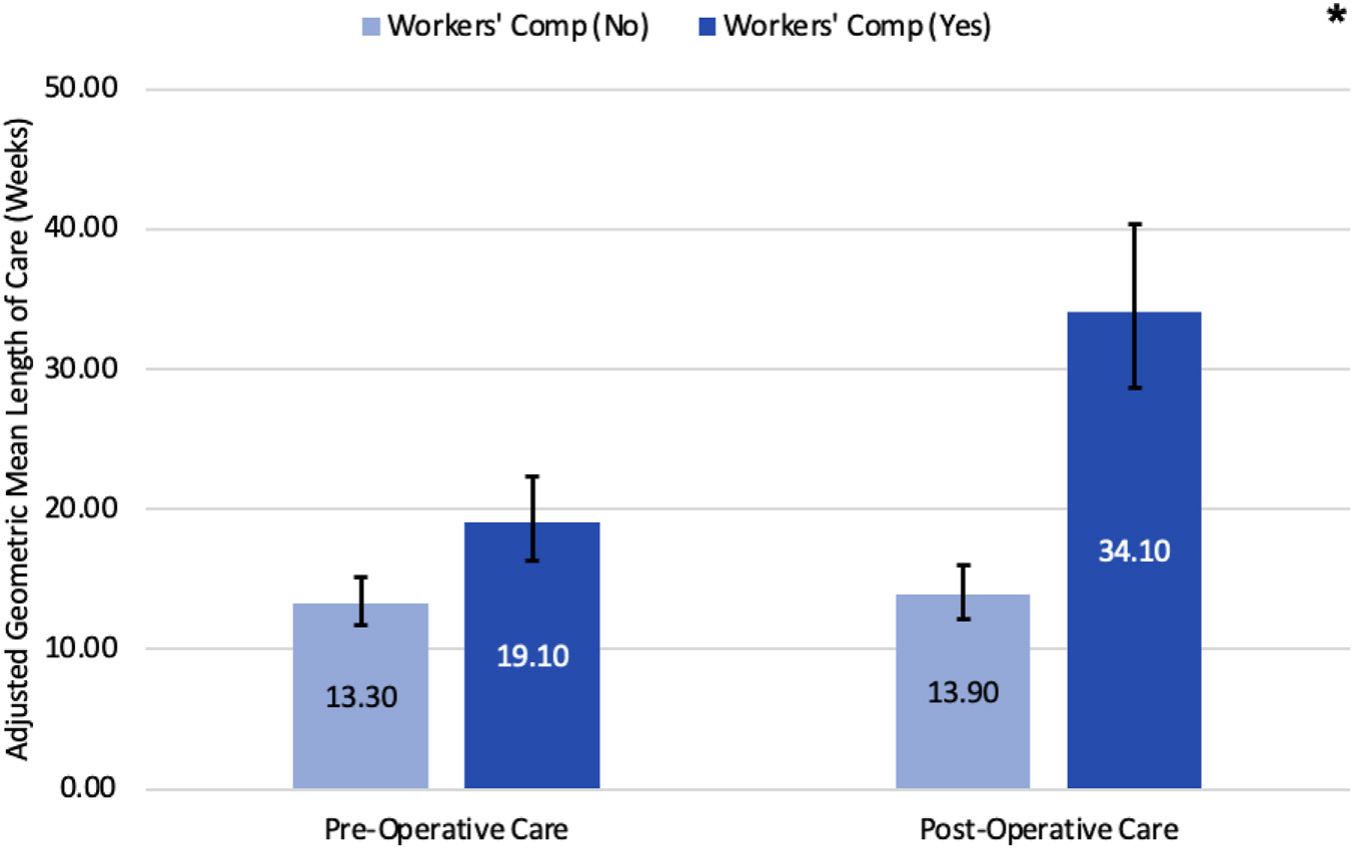
Comparing estimated adjusted geometric means and 95% CI for the interaction between period (preop vs postop) and workers’ compensation regarding length of care (in weeks). Light blue bars represent no workers’ compensation, whereas dark blue represents workers’ compensation. “∗” indicates a statistically significant difference between the two means, with a *P* < .05.

**Figure 6:**
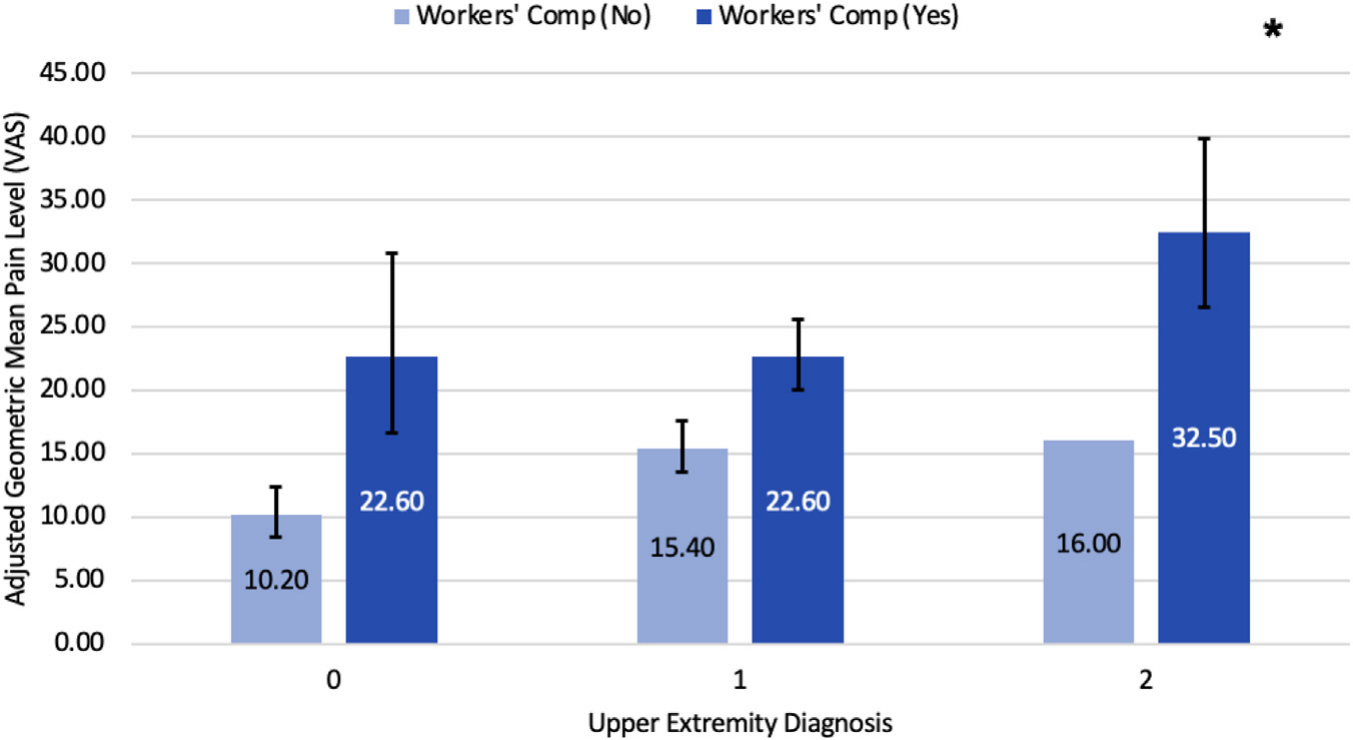
Comparing estimated adjusted geometric means and 95% CI for the interaction between upper extremity diagnosis and workers’ compensation regarding length of care (in weeks). Light blue bars represent no workers’ compensation, whereas dark blue represents workers’ compensation. “∗” indicates a statistically significant difference between the two means, with a *P* value < .05. For the upper extremity diagnosis, 0 = No Additional Upper Extremity Diagnosis, 1 = An Additional Upper Extremity Diagnosis on one side (Unilateral), and 2 = Additional Upper Extremity Diagnoses on both sides (Bilateral).

Adjusted means showed that women, those with traumatic injuries, or rheumatoid arthritis had higher VAS pain scores (Table 6). The interaction between workers compensation and period (pre-op or post-op) showed that the improvement in adjusted mean VAS pain scores from preoperation to postoperation were smaller among those who received workers compensation compared with those who did not (Fig. 4). Regarding length of care, those with an additional upper extremity diagnosis, non-Hispanic black race/ethnicity, cervical spine diagnosis, and workers’ compensation had longer care durations (Table 7). The first interaction showed that those with workers’ compensation had a longer pre- and postoperative length of care (Fig. 5). The second interaction showed that those with an additional upper extremity diagnosis, regardless of bilateral or unilateral involvement, had a longer pre-op and post-op length of care for those with workers compensation when compared to those without workers compensation (Fig. 6). It is noteworthy to mention that trauma did not demonstrate statistical significance in the length of care model.

**Table 6:** Estimated Adjusted Means, 95% CI, and *P* Values Using GEE for VAS Pain Score

Variable	Level	Adjusted Mean	95% CI	*P* Value
Period	Pre-op	6.5	(5.9–7)	< .0001
	Post-op	3.9	(3.3–4.4)	
Gender	Female	5.4	(4.9–6)	.002
	Male	4.9	(4.3–5.5)	
Trauma	No	4.9	(4.4–5.5)	.023
	Yes	5.4	(4.9–5.9)	
Rheumatoid arthritis	No	4.5	(4.3–4.8)	.009
	Yes	5.8	(4.9–6.8)	
Workers’ compensation (WC)	No	4.8	(4.3–5.4)	.001
	Yes	5.5	(4.9–6.1)	
Race/ethnicity	M-A or Hispanic	5.1	(4.4–5.9)	< .0001
	Non-Hispanic Black	5.6	(5.0–6.2)	
	Non-Hispanic White	4.6	(4.1–5.1)	
	Other/unknown	5.3	(4.7–6.0)	
Period∗WC	Pre-op, WC=No	6.3	(5.8–6.9)	.016
	Pre-op, WC=Yes	6.6	(6–7.2)	
	Post-op, WC=No	3.3	(2.8–3.9)	
	Post-op, WC=Yes	4.4	(3.8–5.1)

**Table 7:** Estimated Adjusted Geometric Means, 95% CI, and *P* Values Using GEE for Length of Care

Variable	Level	Adjusted Geometric Mean	95% CI	*P* Value
Period	Pre-op	15.9	(14.3–17.7)	< .0001
	Post-op	21.8	(19.4–24.4)	
Upper extremity (UE) diagnosis	None	15.2	(12.6–18.3)	.0002
	Only one side	18.7	(17–20.5)	
	Both sides	22.8	(19.8–26.3)	
Workers’ compensation (WC)	No	13.6	(12.3–15.1)	< .0001
	Yes	25.5	(22.3–29.2)	
Race/ethnicity	M-A or Hispanic	18.1	(14.8–22)	.0002
	Non-Hispanic Black	22.3	(19.5–25.5)	
	Non-Hispanic White	15.2	(13.4–17.2)	
	Other/unknown	19.6	(16.9–22.8)	
Cervical spine diagnosis	No	16.5	(15–18.1)	.003
	Yes	21.0	(18.3–24.1)	
Period∗WC	Pre-op, WC = No	13.3	(11.7–15.1)	< .0001
	Pre-op, WC = Yes	19.1	(16.3–22.3)	
	Post-op, WC = No	13.9	(12.1–16)	
	Post-op, WC = Yes	34.1	(28.7–40.4)	
UE diagnosis∗WC	None, WC = No	10.2	(8.4–12.4)	.043
	1 side, WC = No	15.4	(13.5–17.6)	
	Both sides∗WC = No	16.0	(13.3–19.2)	
	None, WC = Yes	22.6	(16.6–30.8)	
	1 side, WC = Yes	22.6	(20–25.6)	
	Both sides, WC = Yes	32.5	(26.5–39.9)

## Discussion

This study highlights the relationship between demographic, clinical, and socioeconomic factors that influence pain levels and recovery in patients undergoing carpal tunnel release (CTR). We aimed to compare the characteristics of traumatically induced versus nontraumatically induced CTS to evaluate if these groups differed in reported pain scales, which was measured using a VAS score, or pre- and postoperative length of care. Our results demonstrate that patients with a history of trauma experienced higher initial and final pain scores.

Furthermore, we investigated different demographic and clinical factors that may influence pain scores and length of care. Our findings indicate that workers’ compensation status is a significant predictor of both higher pain levels and longer lengths of care, corroborating prior research showing that individuals on workers’ compensation tend to have poorer postoperative outcomes. This may be a combination of psychological factors and economic stress related to their claim and possible disability. Addressing these factors through tailored counseling could improve recovery rates for this group.^10^^,^^11^

In addition, patients with cervical spine diagnoses experienced longer preoperative care periods and reported higher preoperative pain levels. In the model for length of care, a history of cervical spine diagnosis remained a significant predictor, suggesting that these patients required extended care periods. This is likely because of the contribution of cervical radiculopathy, which can exacerbate symptoms and complicate recovery from CTS surgery. The additional steps needed to rule out cervical spine pathology, such as consultations with spine specialists or obtaining cervical spine X-rays and magnetic resonance imaging, often prolong the process. Effectively managing both cervical spine pathology and CTS is essential to improving both pain management and overall recovery for these patients. Targeted interventions that address both conditions may lead to better long-term outcomes.

A key strength of our study is that all procedures were performed by a single fellowship-trained surgeon, and the postoperative protocol was standardized. However, a significant limitation of this study is the reliance on the self-reported VAS scores, which introduces variability because of individual differences in pain perception and reporting. Additionally, follow-up was somewhat variable, with some patients lost to follow-up rather than formally discharged. On average, patients had 5.4 follow-up visits, with a minimum of zero visits and a maximum of 27 visits.

Another limitation is the retrospective design of the study, which restricts the depth of available data. For example, although we used VAS scores to assess pain, these may not fully capture the complexity of CTS symptoms. The use of condition-specific, validated instruments such as the Boston carpal tunnel questionnaire would provide a more comprehensive assessment of symptom severity and functional limitations, but such data were not available in this retrospective review. Future prospective studies should consider incorporating these measures. The retrospective nature of this study also prevents us from establishing a causal relationship between upper extremity trauma and the development of CTS. Some cases classified as trauma-associated CTS may have been coincidental presentations of idiopathic disease.

The classification of trauma in this study was determined by chart review and may lack specificity in mechanism, anatomical level, or timing. The definition of trauma may therefore be broad or inconsistently applied. We did not quantify the precise time interval between trauma and CTS symptom onset or surgical release, which limits our ability to assess temporal causality. However, inclusion was restricted to cases in which trauma and symptom onset were closely related within the same clinical episode. Also, the use of time from clinic presentation to surgery, and surgery to the final follow-up, as proxies for care duration and recovery does not capture symptom onset or objective functional recovery.

For patients who underwent bilateral CTR, the lack of standardization in the timing between procedures, when performed sequentially, is a potential limitation and may contribute to variability in postoperative recovery and reported outcomes.

In conclusion, this study underscores the importance of considering multiple factors, including trauma history, workers’ compensation status, and comorbidities such as cervical spine disease, when treating CTS. By addressing these factors, clinicians can better tailor treatment strategies and improve surgical outcomes, particularly for those with more complex presentations.

## Declaration of Generative AI and AI-Assisted Technologies in the Writing Process

During the preparation of this work, the author used ChatGPT in order to improve readability and language. After using this tool, the authors reviewed and edited the content as needed and takes full responsibility for the content of the publication.

## Conflicts of Interest

No benefits in any form have been received or will be received related directly to this article.
